# Autophagy-related prognostic signature characterizes tumor microenvironment and predicts response to ferroptosis in gastric cancer

**DOI:** 10.3389/fonc.2022.959337

**Published:** 2022-08-16

**Authors:** Haoran Li, Bing Xu, Jing Du, Yunyi Wu, Fangchun Shao, Yan Gao, Ping Zhang, Junyu Zhou, Xiangmin Tong, Ying Wang, Yanchun Li

**Affiliations:** ^1^ Laboratory Medicine Center, Department of Clinical Laboratory, Zhejiang Provincial People’s Hospital (Affiliated People’s Hospital, Hangzhou Medical College), Hangzhou, China; ^2^ Department of Clinical Laboratory, Hangzhou Women’s Hospital, Hangzhou, China; ^3^ Department of Pulmonary and Critical Care Medicine, Zhejiang Provincial People’s Hospital (Affiliated People’s Hospital, Hangzhou Medical College), Hangzhou, China; ^4^ Department of Central Laboratory, Affiliated Hangzhou First People’s Hospital, Zhejiang University School of Medicine, Hangzhou, China

**Keywords:** autophagy, gastric cancer, tumor microenvironment, microsatellite instability, prognosis, ferroptosis

## Abstract

**Background:**

Gastric cancer (GC) is an important disease and the fifth most common malignancy worldwide. Autophagy is an important process for the turnover of intracellular substances. Autophagy-related genes (ARGs) are crucial in cancer. Accumulating evidence indicates the clinicopathological significance of the tumor microenvironment (TME) in predicting prognosis and treatment efficacy.

**Methods:**

Clinical and gene expression data of GC were obtained from The Cancer Genome Atlas and Gene Expression Omnibus databases. A total of 22 genes with differences in expression and prognosis were screened from 232 ARGs. Three autophagy patterns were identified using an unsupervised clustering algorithm and scored using principal component analysis to predict the value of autophagy in the prognosis of GC patients. Finally, the relationship between autophagy and ferroptosis was validated in gastric cancer cells.

**Results:**

The expression of ARGs showed obvious heterogeneity in GC patients. Three autophagy patterns were identified and used to predict the overall survival of GC patients. These three patterns were well-matched with the immunophenotype. Kyoto Encyclopedia of Genes and Genomes and Gene Ontology enrichment analyses showed that the biological functions of the three autophagy patterns were different. A scoring system was then set up to quantify the autophagy model and further evaluate the response of the patients to the immunotherapy. Patients with high autophagy scores had a more severe tumor mutation burden and better prognosis. High autophagy scores were accompanied by high microsatellite instability. Patients with high autophagy scores had significantly higher PD-L1 expression and increased survival. The experimental results confirmed that the expression of ferroptosis genes was positively correlated with the expression of autophagy genes in different autophagy clusters, and inhibition of autophagy dramatically reversed the decrease in ferroptotic cell death and lipid accumulation.

**Conclusions:**

Autophagy patterns are involved in TME diversity and complexity. Autophagy score can be used as an independent prognostic biomarker in GC patients and to predict the effect of immunotherapy and ferroptosis-based therapy. This might benefit individualized treatment for GC.

## Introduction

Gastric cancer (GC) is the fifth most common cancer worldwide and the third most common cause of cancer-related death. The estimated number of GC cases exceeds one million annually (1). The most important GC risk factor is *Helicobacter pylori* infection; age and a diet high in salt intake are also associated with GC (2–5). GC is usually diagnosed by endoscopy. Surgery or endoscopic resection remains a powerful treatment option (6). GC is mostly found in late stages. Tumor heterogeneity and immune response are due to abnormalities in the tumor microenvironment (TME). Therefore, the prognosis of patients cannot be guaranteed. Although several different molecular classification systems for GC have been proposed in the past decade (7, 8), effective precision treatment strategies still need to be explored.

Autophagy is the process of transporting damaged, denatured, or senescent proteins and organelles in cells to lysosomes for digestion and degradation. Autophagy plays a key role in regulating organismal development and maintaining homeostasis and quality control of proteins and organelles (9). Under normal physiological conditions, autophagy helps cells maintain their homeostatic state (10). During stress, autophagy prevents the accumulation of toxic or carcinogenic proteins and inhibits carcinogenesis. Once a tumor is formed, autophagy provides abundant nutrients for cancer cells and promotes tumor growth (11). Additionally, autophagy is increasingly investigated as a molecular target for cancer therapy. Our recent study demonstrated that excess autophagy results in autophagic cell death (12). However, autophagy plays two roles in tumorigenesis and development. The impact of autophagy on cancer depends on a variety of factors, such as TME, cancer type and stage, and genetic background (13). This reflects the intricate regulatory relationship of autophagy in tumors, which needs to be further clarified through more extensive studies. Ferroptosis is a regulated form of cell death that is morphologically, biochemically, and genetically distinct from apoptosis, necrosis, and autophagy (14). In recent years, research on ferroptosis in cancer has grown rapidly, providing prospects for its application in cancer therapy (15). The interaction between ferroptosis and tumor-related signaling pathways has potential applications in systemic therapy, radiotherapy, and immunotherapy. Targeting GC cells by stimulating ferroptosis through various targets has become a potential therapeutic strategy for gastric cancer (16). In addition, the sensitivity of tumors to ferroptosis therapy has become an important condition for judging the prognosis of patients (15).

The TME is an important component of tumor tissues, including various immune cells, stromal cells, and extracellular components. The composition of resident cell types within the TME and their associated inflammatory pathways differ among cancer patients. These changes correlate with clinical outcomes in various malignancies, including gastric, lung, and breast cancers (17). As malignant tumors develop, they interact with the metabolites of TME, and autophagy is activated in this process to provide nutrients to the tumor (18). Growing evidence indicates the clinicopathological significance of TME in predicting tumor treatment and its prognostic effects (19, 20). Currently, due to technical limitations, only a single autophagy-related gene (ARG) is evaluated in most tumor studies. The characteristics of antitumor mechanisms are by no means explained by one gene, but rather reflect the highly coordinated interaction of numerous genes. Therefore, a comprehensive understanding of the TME mediated by multiple ARGs is required.

In the present study, we identified the role of ARGs in GC progression and predicted the overall survival (OS) of GC patients using a combined analysis of The Cancer Genome Atlas (TCGA) and Gene Expression Omnibus (GEO) databases. Importantly, we report a potential role of cell autophagy patterns in assessing tumor TME, providing important insights for understanding the underlying mechanisms of gastric carcinogenesis and predicting response to immunotherapy and ferroptosis-based therapy.

## Materials and methods

### Data sources

RNA sequencing transcriptome profiling and clinical data of samples, including 343 GC and 30 normal control samples, were downloaded from TCGA database (available online: https://portal.gdc.cancer.gov/). Moreover, GSE84437 (433 samples) with clinical information of stomach adenocarcinoma was downloaded from the GEO database (available online: https://www.ncbi.nlm.nih.gov/geo/). A total of 232 ARGs were obtained from the Human Autophagy Database (available online: http://www.autophagy.lu/index.html). One hundred twenty-one ferroptosis-verified driver genes were obtained from the FerrDb database (available online: http://www.datjar.com:40013/bt2104/), as described previously (21).

### Mutation analysis of ARGs

Mutation frequencies and oncoplot waterfall plots of ARGs in gastric cancer patients were generated by the “maftools” package. The locations of copy number variation (CNV) alterations in ARGs on 23 chromosomes were mapped using the “RCircos” package in R software.

### Identification and functional annotation of differentially expressed genes

Differentially expressed genes (DEGs) between the different autophagy clusters were identified using the “limma” package in R with an adjusted p-value of <0.001. Kyoto Encyclopedia of Genes and Genomes (KEGG) analysis and Gene Ontology (GO), including biological process (BP), cellular component (CC), and molecular function (MF) categories, were performed with the “ggplot2” package in R software to further explore the potential functions of autophagy-related DEGs (22).

### Immune infiltration, tumor mutation burden, and microsatellite instability analysis

We used the ssGSEA (single-sample gene-set enrichment analysis) algorithm to quantify the relative abundance of each cell infiltration in the gastric cancer TME. Correlations between prognostic ARGs and immune filtering were analyzed using a TME-filtered immune cell gene set with diverse human immune cell subtypes, including activated CD8 T cells, activated dendritic cells, giant natural killer T cells, and regulatory T cells. In tumor mutation burden (TMB) and microsatellite instability (MSI) analyses, Spearman correlation analysis was used to calculate the correlation between high- and low-autophagy score groups. MSI status was classified as microsatellite stable (MSS), MSI-low (MSI-L, one marker unstable), and MSI-high (MSI-H, over two markers unstable).

### The establishment of an autophagy scoring model and prognostic analysis

Principal component analysis (PCA) was used to evaluate the autophagy gene signature of each gastric cancer patient, which we termed as autophagy score. Patients were divided into the high-score group and low-score group based on the maximally selected rank statistics determined by the “survminer” R package. We used Kaplan–Meier survival curves to identify the ability of the model to distinguish different clusters of patients to determine the efficiency of the model.

### Cell viability assay

BGC823 cells were seeded into 96-well plates in DMEM (Gibco, Carlsbad, CA, USA), supplemented with 10% fetal bovine serum (Gibco, USA), 100 U/ml penicillin, and 100 μg/ml streptomycin at a density of 2 × 10^4^ cells/well. Cells were treated with bafilomycin A1 (BafA1, 40 nm), chloroquine (CQ, 25 µM), and 3-methyladenine (3MA, 2 mM) in the presence or absence of erastin for 36 h. Then, 10 μl of Cell Counting Kit-8 (Beyotime) reagent was added to each well and incubated for 2 h at 37°C. The absorbency was measured at 450 nm using a plate reader, and the percentage viability was calculated.

### BODIPY staining

BGC823 cells in culture were collected in a chamber confocal dish and incubated with boron dipyromethene (BODIPY 581/591) (Thermo Fisher Scientific) at a concentration of 5 μM, and nuclei were counterstained with DAPI for 10 min. Quantification of lipid bodies was performed using ImageJ.

### Ethics statement

Ethical approval was not required, as there is no patient recruitment and absence of animal trials for this study.

### Statistical analyses

The correlation coefficient between TME and ARG expression in filtered immune cells was calculated by Spearman and differential expression analyses. Continuous variables were compared between two groups through the Wilcoxon rank-sum test. Classified variables were compared between two groups by chi-square test. One-way ANOVA and Kruskal–Wallis test were used to conduct difference comparisons of three or more groups. The R package of “survminer” was used to determine the cutoff point for each dataset subgroup. The survival curves for the prognostic analysis were generated *via* the Kaplan–Meier method, and log-rank tests were utilized to identify the significance of differences. The waterfall function of the maftools package was used to present the mutation landscape in patients with high and low autophagy scores in TCGA-STAD cohort. All statistical analyses were performed using R version 4.1.0. Statistical significance was set at p < 0.05.

## Results

### Defining expression of ARGs in GC

**Figure 1:**
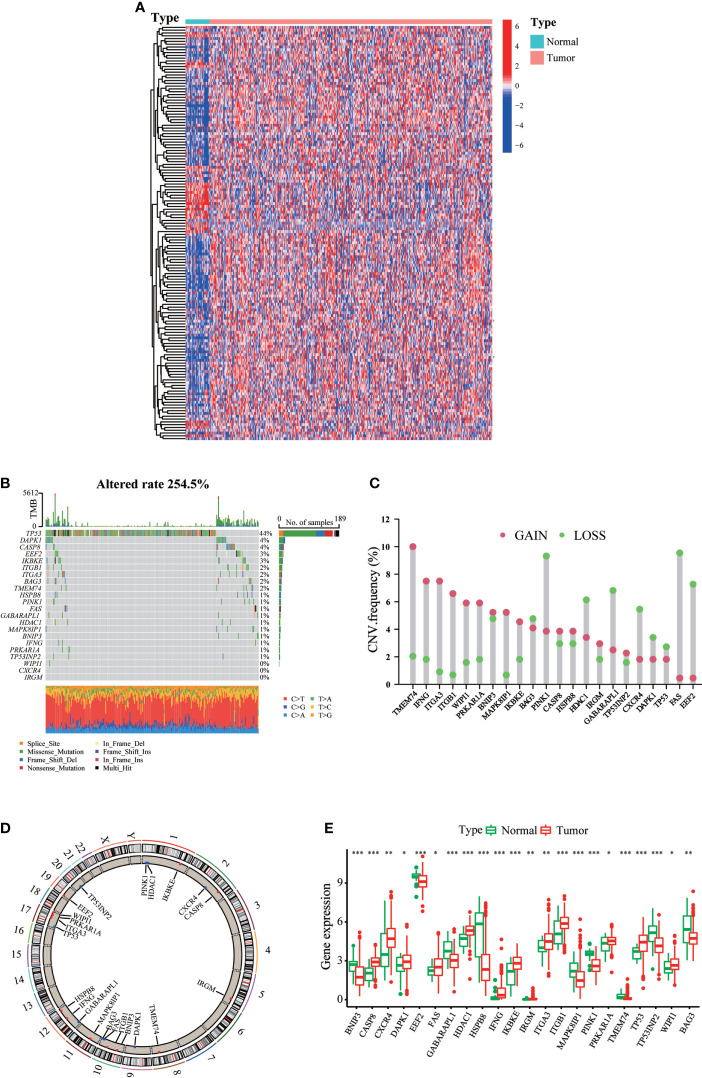
Landscape of genetic and expression variation of ARGs in gastric cancer. **(A)** mRNA expression profiles of 148 differentially expressed ARGs in TCGA-STAD cohort. **(B)** The mutation frequency of ARGs in gastric cancer patients of TCGA-STAD cohort. The upper bar graph shows TMB; the right bar graph shows the proportion of each variant type. **(C)** The CNV variation frequency of each ARG based on CNV variation. **(D)** The location of CNV alteration of ARGs on 23 chromosomes. **(E)** Expression distributions of ARGs between normal (green) and tumor (red) tissues. ^∗^p < 0.05; ^∗∗^p < 0.01; ^∗∗∗^p < 0.001.

A total of 232 ARGs were obtained from the Human Autophagy database. A heatmap involving TCGA-STAD cohort revealed differences in the expression profiles of 148 ARGs in normal and tumor tissues (**Figure 1A**). Univariate Cox regression analysis of these DEGs revealed that 22 ARGs were significantly associated with TCGA-STAD prognosis (**Supplementary Table 1**). **Figure 1B** presents the incidence of somatic mutations in the 22 ARGs in GC. The TP53 gene displayed the highest mutation frequency (44%), followed by the DAPK1 and CASP8 genes, among the 22 ARGs. We considered the relationship between TP53 mutations and ARG expression, in light of the highest mutation frequency in TP53. The expression levels of eight ARGs were significantly correlated with TP53 mutation status (**Figure S1**). We then investigated somatic copy number alterations in these ARGs. Copy number changes were evident in all 22 ARGs. More than half of the 22 ARGs had copy number amplification, while CNV deletion frequencies, such as BAG3 and PINK1, were widespread (**Figure 1C**). **Figure 1D** shows the location of CNV alterations in these ARGs on the chromosomes. We further compared the mRNA expression levels between GC and normal tissues. The expression of 13 ARGs was increased, while the expression of nine ARGs was decreased in tumors compared with normal tissues in GC (**Figure 1E**). More specifically, compared to normal tissues, the expression of CNV-increased autophagy modulators in GC tissues (such as CASP8 and CXCR4) was significantly increased. Conversely, the expression of CNV-deficient autophagy modulators of GC tissues (such as BNIP3 and EEF2) was reduced, suggesting that CNV variation may be a cause for the regulation of ARG expression. Additionally, the expression levels of 22 ARGs were associated with survival in GC patients (**Figure S2**), suggesting that ARGs were involved in the development of GC and had the potential to predict patient prognosis.

### Identification of the autophagy clusters

**Figure 2:**
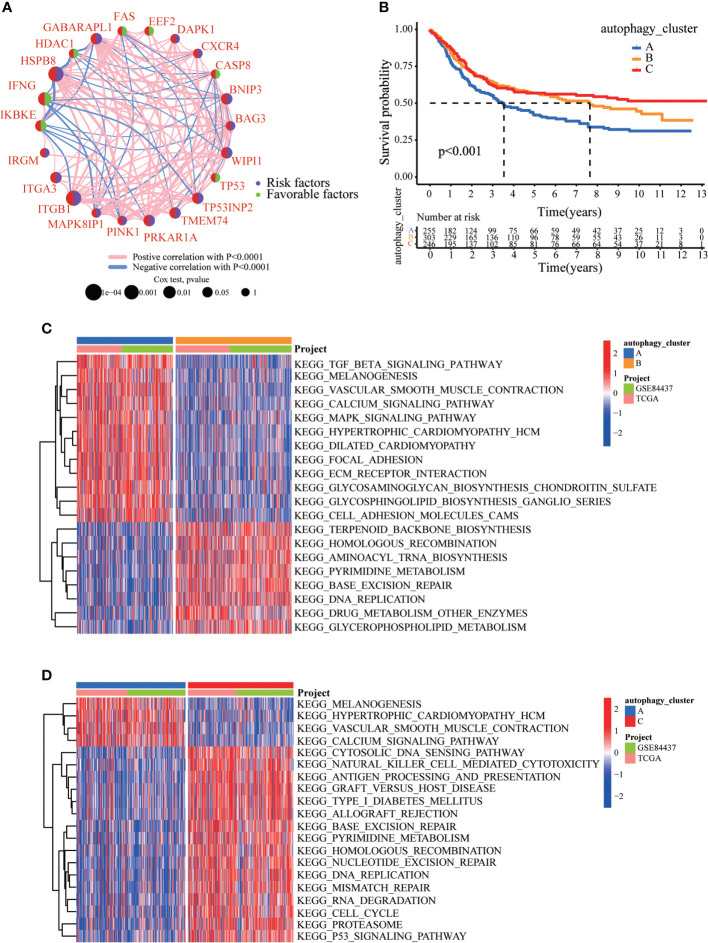
The clinicopathological and biological characteristics of three autophagy clusters. **(A)** Interaction network diagram between the ARGs. **(B)** Survival analysis for the three autophagy clusters in gastric cancer patients. **(C)** The differences between functional pathways in autophagy clusters by GSVA. Blue represents the autophagy cluster A, and orange represents the autophagy cluster B. **(D)** The differences between functional pathways in autophagy clusters by GSVA. Orange represents the autophagy cluster B, and red represents the autophagy cluster C.

We created a queue using the GEO and TCGA datasets along with OS data and clinical information to construct a more precise autophagy cluster with prognostic significance. The autophagy network diagram showed that the expression of most ARGs was positively correlated, with some genes being negatively correlated (**Figure 2A**). Subsequently, we identified three different regulation patterns using the unsupervised clustering method (**Figure S3**). The survival advantage of clusters B and C was higher than that of cluster A (**Figure 2B**).

### Infiltration characteristics of TME cells under different autophagy modification patterns

**Figure 3:**
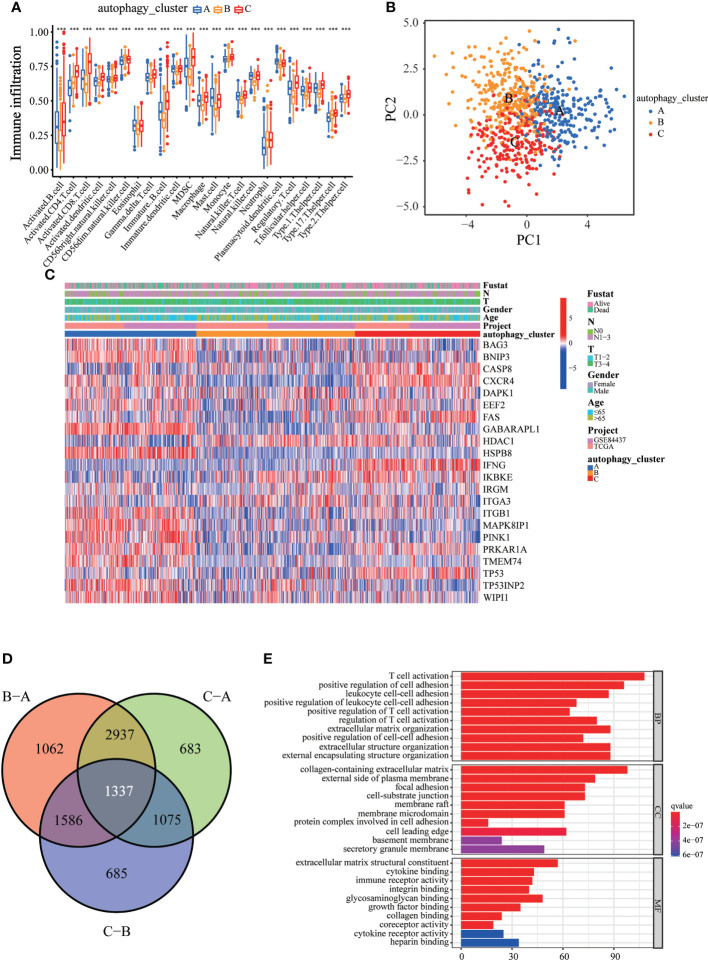
Immune cell infiltration and transcriptome features among the three autophagy clusters. **(A)** The differences in immune cell infiltration among three autophagy clusters. **(B)** The scatter plot of principal component analysis. **(C)** Clinicopathological features and expression levels of 22 ARGs in three autophagy clusters. **(D)** Venn diagram showing overlapping genes in three autophagy clusters. **(E)** GO enrichment analysis of the overlapping genes. ^∗∗∗^p < 0.001.

To explore the differences in biological behavior among these three patterns, we performed GSVA enrichment analysis (**Figures 2C, D**). Autophagy cluster A was markedly enriched in stromal and carcinogenic activation pathways, such as TGFβ signaling pathway, ECM receptor interaction, cell adhesion, and MAPK signaling pathways. Autophagy cluster B was significantly associated with biological metabolism. Autophagy cluster C presented enrichment pathways associated with immune full activation, including the activation of chemokine signaling, JAK-STAT signaling, T-cell receptor signaling, and Toll-like receptor signaling pathways. Subsequent analysis of TME cell infiltration showed that autophagy cluster C was remarkably rich in innate immune cell infiltration, including natural killer cells, macrophages, MDSCs, monocytes, and immature B cells. The three autophagy modification patterns showed significantly different infiltration characteristics of TME cells (**Figure 3A**). PCA revealed significant differences in the autophagy modification profiles between the three subtypes (**Figure 3B**). Comparison of the clinicopathological features of GC revealed significant differences in the expression of ARGs and clinicopathological characteristics (**Figure 3C**). Among these autophagy-related DEGs, the intersection of the three autophagy clusters A, B, and C confirmed 1,337 DEGs (**Figure 3D**). To clarify the function of these DEGs, pathways were analyzed using GO and KEGG databases. The 1,137 DEGs were mainly involved in T-cell activation, positive regulation of cell adhesion, extracellular matrix organization, extracellular structure organization, and collagen-containing extracellular matrix (**Figure 3E** and **S4A**). Moreover, KEGG pathway analysis suggested that these DEGs were mainly involved in cell adhesion molecules, cytokine–cytokine receptor interactions, cell adhesion molecules, chemokine signaling pathways, and focal adhesion (**Figures S4B, C**). Then, 632 differentially expressed and prognostic genes were screened out from the three autophagy clusters and used for the subsequent analysis.

### Construction of autophagy gene signature and functional annotation

**Figure 4:**
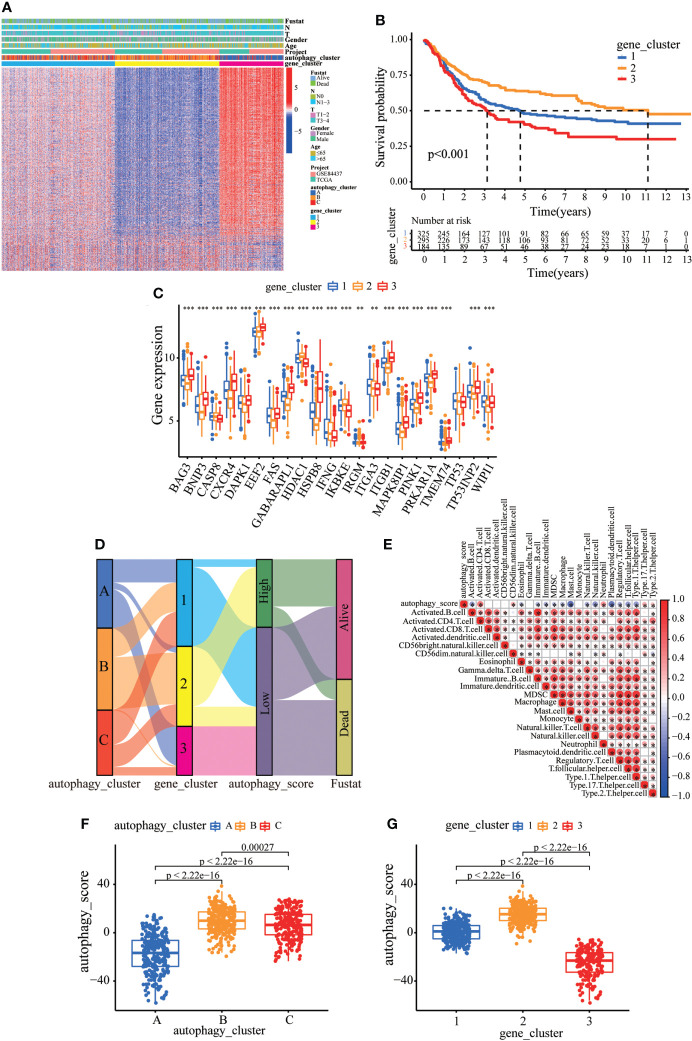
Construction of the autophagy score. **(A)** Heatmap of relationships between clinicopathological features and autophagy gene clusters. **(B)** Kaplan–Meier survival curves of different autophagy gene clusters. **(C)** The differential expression of ARGs among different gene clusters. **(D)** Alluvial diagram showing genotype distributions in different gene clusters and survival outcomes. **(E)** The correlation analysis between the autophagy score and immune cells; red indicates positive correlation, and blue indicates negative correlation. **(F)** Differences in autophagy score among three autophagy clusters. **(G)** Differences in autophagy score among three gene clusters. ^∗∗^p < 0.01; ^∗∗∗^p < 0.001.

Consistent with the clustering grouping of autophagy modification patterns, the unsupervised clustering algorithm also divided the patients into three distinct autophagy modification genomic phenotypes depending on the 632 prognostic genes (gene clusters 1, 2, and 3; **Figure S5**). The heat map of the genetic modification patterns revealed that most genes were expressed at low levels in gene cluster B and were highly expressed in gene cluster C (**Figure 4A**). The findings indicate the presence of three distinct autophagy modification patterns in GC. Kaplan–Meier curves showed that patients with gene cluster 3 had the worst prognosis, whereas patients in cluster 2 showed a favorable prognosis (**Figure 4B**). The three autophagy gene clusters showed significant differences in the expression of ARGs, consistent with the three autophagy clusters (**Figure 4C**). Considering the individual heterogeneity and complexity of autophagy, we developed an autophagy score based on principal component analysis to quantify autophagy modification patterns in individual GC patients. Then, we divided the patients into high-score group and low-score group. An alluvial diagram was used to visualize the flow of the autophagy score fraction construction (**Figure 4D**). We then conducted immune correlation analysis. The autophagy score was significantly positively correlated with CD4 T immune cells and neutrophils and negatively correlated with activated B immune cells and macrophages (**Figure 4E**). Differences were evident in autophagy scores among the autophagy clusters and also among the three gene clusters. Autophagy cluster A showed the lowest score compared with the other clusters (**Figure 4F**). Simultaneously, compared with the other clusters, autophagy gene cluster 2 had the highest autophagy score and autophagy gene cluster 3 had the lowest score (**Figure 4G**).

### Autophagy molecular subtypes and prognosis

**Figure 5:**
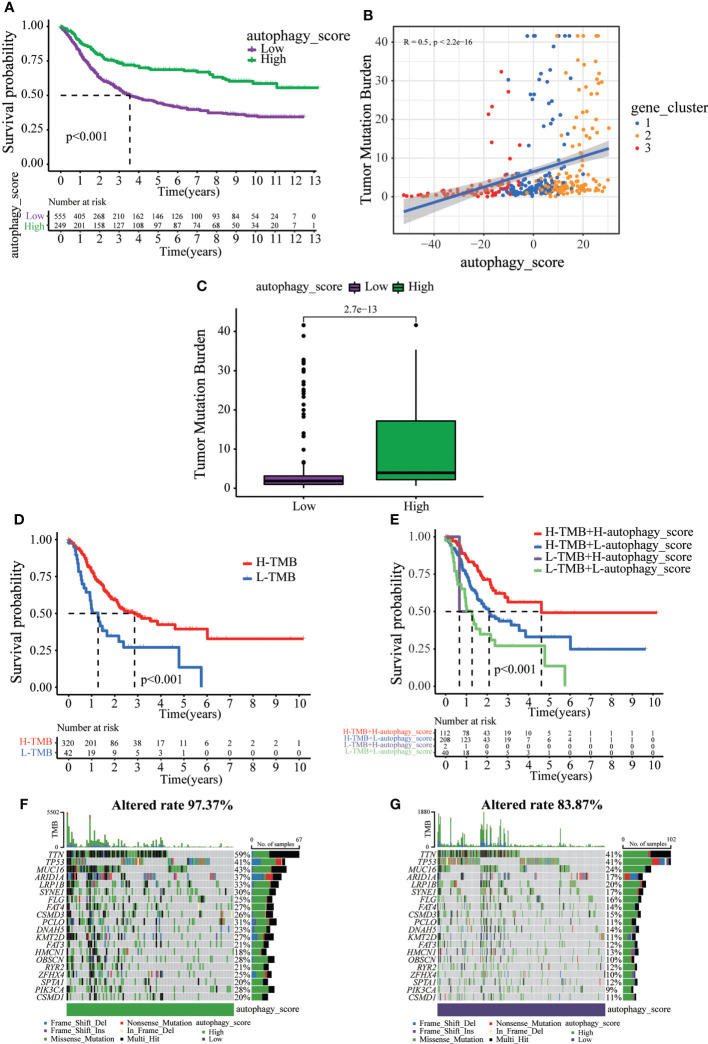
The relationship between autophagy score and tumor somatic mutation. **(A)** Survival analysis of the patients with high autophagy scores and low autophagy scores. **(B)** Spearman correlation analysis of the autophagy score and TMB. **(C)** TMB in different autophagy score groups. **(D)** Survival analysis of low or high TMB in gastric cancer patients. **(E)** Survival analysis of TMB combined with autophagy score in gastric cancer patients. **(F)** The waterfall plot of somatic mutation features established with high autophagy score. **(G)** The waterfall plot of somatic mutation features established with low autophagy score.

Next, we tried to further determine the value of autophagy score in predicting the prognosis of GC patients. The prognosis of patients in the low autophagy score group was poorer than that of patients in the high autophagy score group (**Figure 5A**). In addition, Spearman correlation analysis demonstrated that the autophagy score was positively associated with TMB, which reflects the total number of mutations carried by tumor cells and is related to tumor recognition by immune cells (**Figure 5B**). We explored the association of TMB with different autophagy scores. TMB in the high autophagy score group was greater, indicating a better response to immunotherapy (**Figure 5C**). Survival analysis of TMB in GC revealed that the prognosis of the high-TMB group was better than that of the low-TMB group (**Figure 5D**). As expected, the TMB survival curve combined with the autophagy score showed that patients in the high tumor mutation group and high autophagy score group had the best prognosis (**Figure 5E**). As shown in **Figures 5F, G**, the high autophagy score group had a higher TMB frequency than the low autophagy score group (total genes rate 97.37% versus 83.87%). These results indicate the value of the autophagy score in predicting the prognosis of GC patients and reflect the effect of immunotherapy to a certain extent.

### Role of autophagy score in GC immunotherapy and chemotherapy

**Figure 6:**
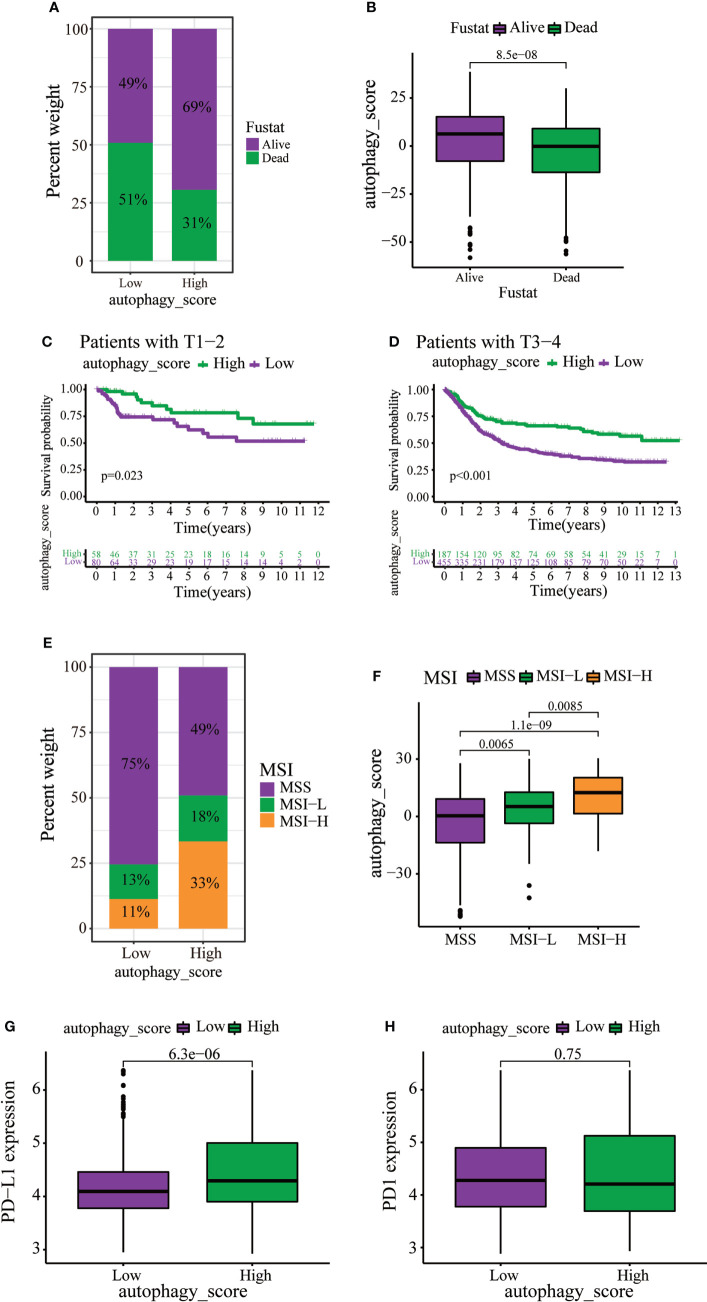
The role of autophagy score in immunotherapy and chemotherapy. **(A, B)** Stratified analysis of autophagy scores in gastric cancer patients according to survival status. **(C, D)** Stratified analysis of autophagy scores in gastric patients according to the T stage. **(E)** Relationships between autophagy score and MSI. **(F)** Stratified analysis of autophagy scores in gastric patients according to MSI. **(G, H)** Expression levels of PD-L1 and PD1 in two distinct groups.

Immunotherapy can increase the survival rate of patients with multiple types of tumors. Therefore, it is important to determine which patients could respond better to immunotherapy. Survival analysis revealed that death of GC patients occurred mainly in the low autophagy score group (**Figure 6A**). Moreover, the autophagy score was lower in patients who died of GC (**Figure 6B**). Stratified analysis of the autophagy score for the GC patients showed that the high autophagy score group had a better prognosis than the low autophagy score group of T1–2 and T3–4 (**Figures 6C, D**). MSI has been associated with the development of tumors. MSI-high (MSI-H) patients are more sensitive to immunotherapy (23). In the present study, the high autophagy score was accompanied by the MSI-H state, while a low autophagy score was accompanied by a microsatellite stable state (**Figures 6E, F**). Immunotherapy targeting PD1 and PD-L1 has improved survival in cancer (24). In this study, GC patients with high autophagy scores displayed significantly high PD-L1 expression, suggesting a potential benefit of anti-PD-L1 immunotherapy (**Figures 6G, H**).

### Ferroptosis in different autophagy subtypes in GC patients

**Figure 7:**
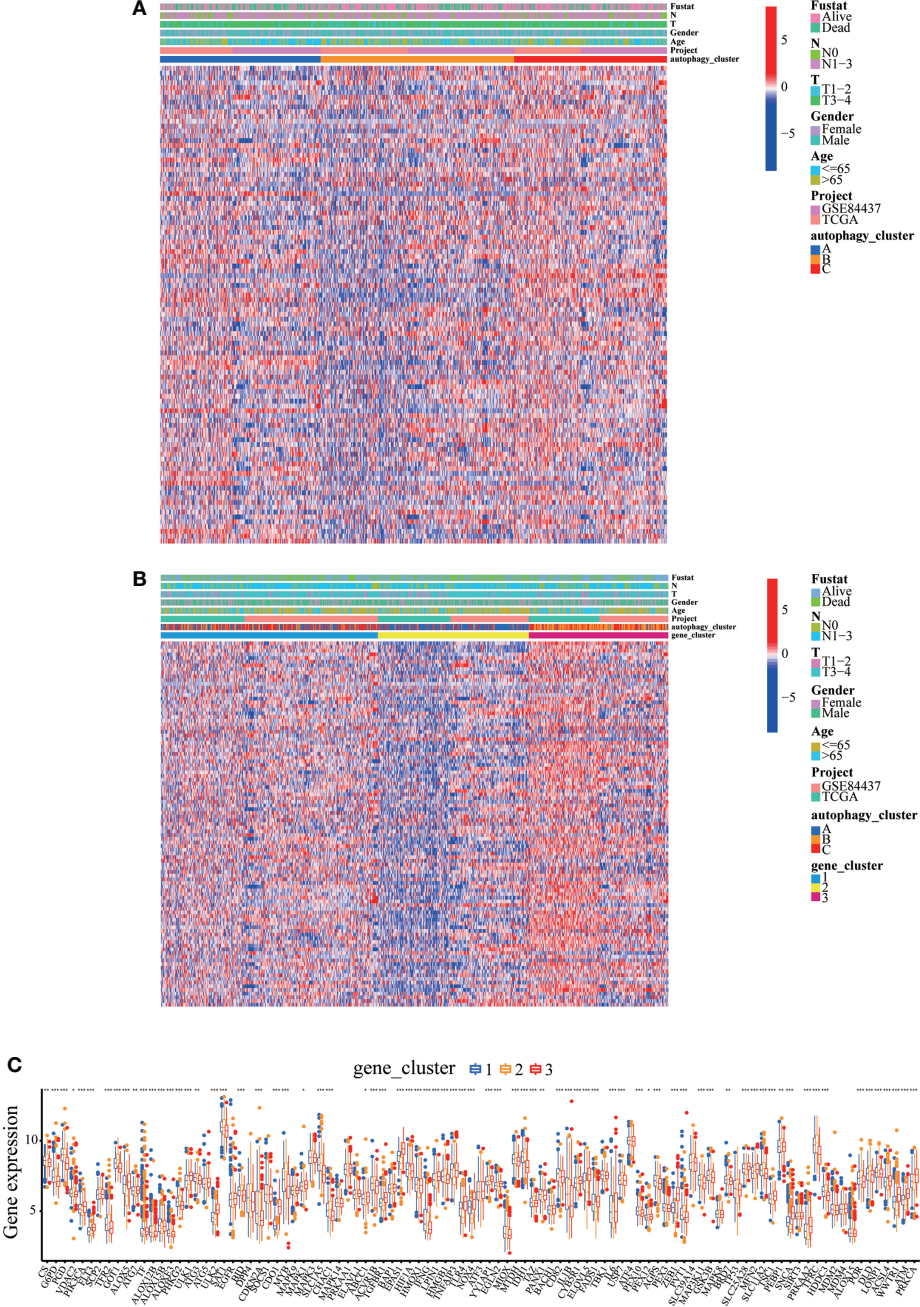
Expression levels of ferroptosis-verified driver genes in different autophagy patterns. **(A)** Clinicopathological features and expression levels of 121 ferroptosis-verified driver genes in three autophagy clusters. **(B)** Clinicopathological features and expression levels of 121 ferroptosis-verified driver genes in three autophagy gene clusters. **(C)** The differential expression of ferroptosis-verified driver genes among different gene clusters. *p<0.05, **p<0.01, ***<p.0.001.

Ferroptosis, a novel form of regulated cell death, is associated with iron accumulation and lipid peroxidation (25, 26). Our recent studies demonstrated that achieving ferroptosis *via* ferroptosis-inducing drugs is emerging as a new alternative therapy modality (27–29). Moreover, in our previous study, autophagy accelerates the degradation of ferritin, increases the unstable iron pool, promotes the accumulation of cellular reactive oxygen species, and ultimately leads to ferroptosis (30). Therefore, we extracted 121 ferroptosis-verified driver genes from the FerrDb database and analyzed the association of these genes in our established autophagy model in GC patients. As expected, in GC patients, these ferroptosis-verified driver genes showed differential expression in different autophagy clusters (**Figure 7A**). Surprisingly, the heat map showed that ferroptosis-verified driver genes were reduced in gene cluster B and highly expressed in gene cluster C, which was consistent with the expression level of ARG (**Figures 4A** and **7B**). In addition, three gene clusters showed significant differences in the expression of ferroptosis-verified driver genes (**Figure 7C**). We can conclude that the expression of genes related to ferroptosis was positively correlated with the expression of ARGs.

### Validation of functional phenotypes in GC cell lines

**Figure 8:**
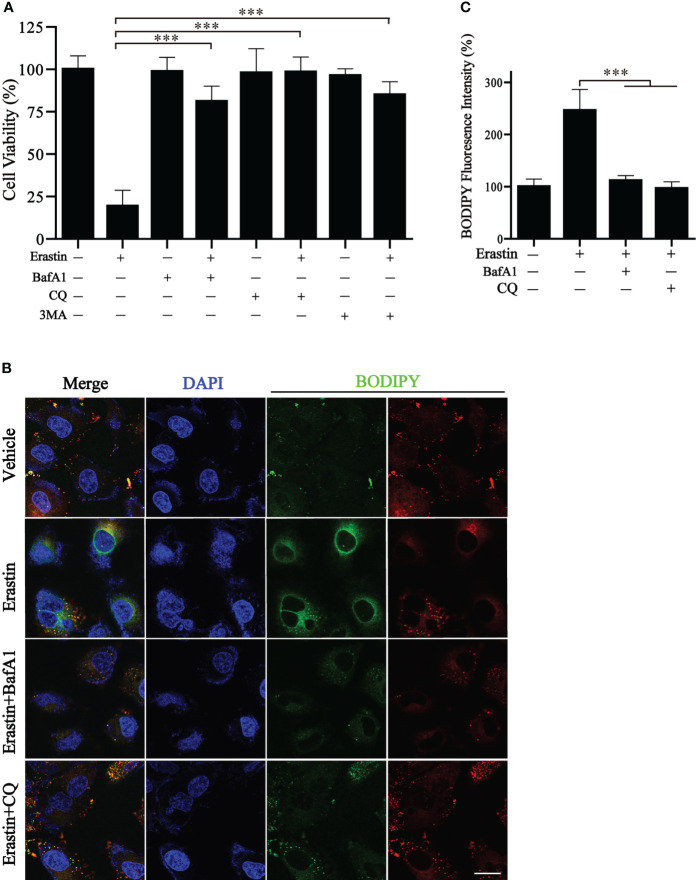
Inhibition of autophagy blocks erastin-induced ferroptosis. **(A)** Cell viability of BGC823 cells with the treatment of erastin in the presence or absence of autophagy inhibitors. **(B, C)** Representative BODIPY fluorescence images with indicated treatment and respective quantification. Scale bars = 10 μm. Data represent mean ± SEM. ^***^p < 0.001 vs. erastin-treated cells.

We wonder whether the autophagy cluster model could predict the sensitivity to ferroptosis-inducing therapy. BGC823 cells were induced to undergo ferroptosis with erastin *in vitro*. Of note, a significant reduction in cell viability by erastin treatment was observed, but cell viability was significantly reversed by different autophagy inhibitors, including BafA1, CQ, and 3MA (**Figure 8A**). We also detected the generation of lipid reactive oxygen species (ROS) by BODIPY, a classical ferroptosis maker (16). The fluorescence results showed a large amount of lipid ROS accumulation in BGC823 cells under the treatment of erastin, while the presence of autophagy inhibitors dramatically ameliorated the accumulation of lipid ROS (**Figures 8B, C**). These results suggest that detection of autophagy typing can predict tumor susceptibility to ferroptosis therapy.

## Discussion

Growing evidence suggests that autophagy plays an integral role in inflammation, innate immunity, and antitumor activity by degrading damaged organelles and excess proteins (31, 32). Autophagy has various roles in various cancers. Historically, the role of autophagy in tumorigenesis may have been misunderstood. The clinical use of autophagy inhibitors may not have a positive effect on cancer patients but may promote tumorigenesis (33). Little is known about the phenomenon of autophagy in GC cells, and the mechanism between autophagy and GC remains controversial. However, studies in animal models have shown that the inhibitory effect of autophagy on tumors may be greater than its facilitation in cells with impaired apoptotic machinery (34). In this study, we identified 22 ARGs and classified them into three clusters. Moreover, combining the filtering properties of TME cells in different clusters of ARGs generated data that improve the understanding of TME antitumor immune responses in GC.

We observed that the three clusters of autophagy patterns were significantly correlated with immune activation and other pathways. Cluster A was characterized by immunosuppression, corresponding to the immune desert phenotype. Cluster B was characterized by the activation of innate immunity and matrix, corresponding to an immune-excluded phenotype. Cluster C was characterized by the activation of adaptive immunity, corresponding to the immune inflammatory phenotype. The latter phenotype corresponds to the “hot tumor”, in which CD4 and CD8 T cells are expressed in the tumor parenchyma. The immune-excluded phenotype has abundant immune cells that do not penetrate the parenchyma of these tumors but which remain in the matrix surrounding the tumor cells. The immune desert phenotype corresponds to the “cold tumor”, with no T cells in the tumor parenchyma or stroma (35, 36). Our results were consistent with these definitions, confirming that different patterns of autophagy are important in shaping the antitumor immune response in different TME landscapes. Cluster C featured the activation of chemokines, T-cell receptors, and Toll-like receptor signaling pathways. All these pathways contribute to the involvement of cluster C in immune inflammation typing. Therefore, it was not surprising that cluster C activated innate immunity and resulted in a better survival curve.

Similar to the clustering results of the three modes of autophagy, three gene clusters were identified based on the DEGs among the three autophagy clusters, which were also significantly associated with stroma and immune activation. This confirmed that autophagy is involved in the composition and structure of the TME landscape. Therefore, analysis of autophagy patterns will help understand the characteristics of TME cell infiltration. In this study, we established a scoring system to assess autophagy patterns in patients with GC. Autophagy scores were higher for the autophagy patterns of the immune-excluded phenotype. The autophagy score was significantly positively correlated with CD4 T immune cells, neutrophils, and macrophages, suggesting that the autophagy score could be used to assess tumor autophagy patterns and immunophenotypes. In addition, the gene mutation frequency in the high autophagy score group was higher than the total gene mutation frequency in the low autophagy score group. Patients in the high autophagy score group also had better survival rates across the different cancer stages. Furthermore, we found that autophagy patterns influenced the therapeutic effect of the immune checkpoint blockade. The autophagy score was markedly correlated with MSI status and PD-L1 expression, which might be a more effective predictor of immunotherapy.

Previous studies have demonstrated that Beclin1, LC3, and P62/SQSTM1 are autophagy-related markers with prognostic values in GC (37–39). Compared with normal mucosal epithelial cells, the expression of BNIP3 is increased in malignant gastric epithelial cells than in normal mucosal epithelial cells, suggesting that BNIP3 expression may play a role in GC development (40). However, the molecular mechanisms of many other ARGs in GC are not yet fully understood. Therefore, considering ARGs as a whole to construct a tumor prediction model will be an effective method to study autophagy and tumor development. Assessing tumor-driver mutations is a key basis for cancer diagnosis and treatment (41). We observed that patients with high autophagy scores had significantly higher frequencies of TTN, MUC16, and ARID1A mutations than patients with low autophagy scores. Moreover, the TTN mutation spectrum serves as a predictor of MSI-H and the mutational load in the TTN also represents a high TMB state (42). In the present study, the proportion of patients with MSI-H was higher in those with high autophagy scores. This suggests a complex interplay between autophagy patterns and immune genes in TMB.

The concept of ferroptosis-suppressing tumors has become widely accepted, with FDA-approved drugs identified as ferroptosis inducers and the potential of ferroptosis as a new promising approach to killing therapy-resistant cancers (43). Past studies have emphasized that the regulation of ferroptosis is autophagy-dependent and involves multiple autophagy-related molecular factors in the process of ferroptosis (44). Our results found that the expression of ferroptosis genes was positively correlated with the expression of autophagy genes in GC patients. Furthermore, inhibition of autophagy significantly reversed the decline in cell viability and lipid accumulation caused by ferroptosis. Therefore, we have reason to believe that our established autophagy analysis can predict the sensitivity of GC patients to ferroptosis treatment.

This study has some limitations that need to be acknowledged. As all analyses were based on data from public databases, extensive *in vivo* and *in vitro* experiments are still required to support our findings. Thus, further studies should be performed to prove the relationship between autophagy and GC in the future.

In conclusion, we performed comprehensive and systematic bioinformatics analyses of GC patients and identified 22 ARGs to analyze their application in GC. The findings establish an autophagy scoring system for GC patients. Our findings concerning the association between autophagy score and clinicopathological features indicate that the autophagy score could serve as an independent prognostic biomarker in GC patients. The autophagy score can also predict the effect of immunotherapy and ferroptosis-based treatment in GC patients, providing new insights for guiding the precise treatment of such patients.

## Data availability statement

The original contributions presented in the study are included in the article/**Supplementary Material**. Further inquiries can be directed to the corresponding authors.

## Author contributions

YL conceptualized and refined this work. YW designed *in vitro* experiments, XT designed the bioinformatics experiments. HL, BX, and JD analyzed the results and drafted the manuscript. HL, YYW, FS, and YG compiled the data. BX, JD, PZ, and JZ combined and examined the data. All authors read and approved the final manuscript.

## Funding

This research was supported by the National Natural Science Foundation of China (No. 82102938), Zhejiang Public Welfare Technology Application Research Project (Grant Nos. LGF19H080006, LGF21H010008, LGF20H080005), and Medical and Health Science and Technology Project of Zhejiang Province (Nos. 2021KY842, 2021KY483, 2021KY077, 2022KY503, 2022KY046, 2022KY236, 2022KY074).

## Acknowledgments

The authors would like to acknowledge TCGA and GEO databases for their assistance, and the contributors for uploading valuable and meaningful resources.

## Conflict of interest

The authors declare that the research was conducted in the absence of any commercial or financial relationships that could be construed as a potential conflict of interest.

## Publisher’s note

All claims expressed in this article are solely those of the authors and do not necessarily represent those of their affiliated organizations, or those of the publisher, the editors and the reviewers. Any product that may be evaluated in this article, or claim that may be made by its manufacturer, is not guaranteed or endorsed by the publisher.
