# Supplementary material for: Autophagy-related prognostic signature characterizes tumor microenvironment and predicts response to ferroptosis in gastric cancer
Source: Front Oncol. 2022 Aug 16;12:959337. doi: 10.3389/fonc.2022.959337 (PMC9424910; doi:10.3389/fonc.2022.959337)
Supplement: Supplementary file 1 [file DataSheet_1.docx]

Supplementary Material

## Supplementary Table

**Table S1. The identified 22 ARGs correlated with prognosis**

| **Gene** | **HR** | **HR.95L** | **HR.95H** | **p-value** |
| --- | --- | --- | --- | --- |
| BAG3 | 1.16068887 | 1.01522956 | 1.3269892 | 0.02916802 |
| BNIP3 | 1.15358443 | 1.05231984 | 1.2645937 | 0.00230477 |
| CASP8 | 0.72898999 | 0.56981174 | 0.93263505 | 0.01190939 |
| CXCR4 | 1.0951121 | 1.00225786 | 1.19656883 | 0.04444664 |
| DAPK1 | 1.13895186 | 1.00987073 | 1.28453207 | 0.03400512 |
| EEF2 | 0.82946949 | 0.69042596 | 0.99651473 | 0.04579697 |
| FAS | 0.87921747 | 0.77434689 | 0.99829077 | 0.04699312 |
| GABARAPL1 | 1.22380048 | 1.07736956 | 1.39013359 | 0.00189576 |
| HDAC1 | 0.81481274 | 0.67588132 | 0.98230232 | 0.03177934 |
| HSPB8 | 1.14173415 | 1.07304044 | 1.21482547 | 2.83E-05 |
| IFNG | 0.81419057 | 0.7298834 | 0.90823588 | 0.000228 |
| IKBKE | 0.7990146 | 0.68759846 | 0.92848423 | 0.00340719 |
| IRGM | 1.73377134 | 1.07512051 | 2.79593129 | 0.02400518 |
| ITGA3 | 1.19681346 | 1.06513743 | 1.34476775 | 0.00251883 |
| ITGB1 | 1.44971168 | 1.23588966 | 1.70052719 | 5.08E-06 |
| MAPK8IP1 | 1.15148833 | 1.02290059 | 1.2962407 | 0.01955712 |
| PINK1 | 1.17237684 | 1.00059457 | 1.37365073 | 0.04914669 |
| PRKAR1A | 1.27136785 | 1.0706523 | 1.50971161 | 0.00616865 |
| TMEM74 | 1.49333715 | 1.11453365 | 2.00088695 | 0.00722324 |
| TP53 | 0.87758986 | 0.78275684 | 0.98391214 | 0.02522528 |
| TP53INP2 | 1.21211778 | 1.05875717 | 1.38769261 | 0.00531625 |
| WIPI1 | 1.20331655 | 1.04882594 | 1.38056341 | 0.00829232 |

## Supplementary Figures


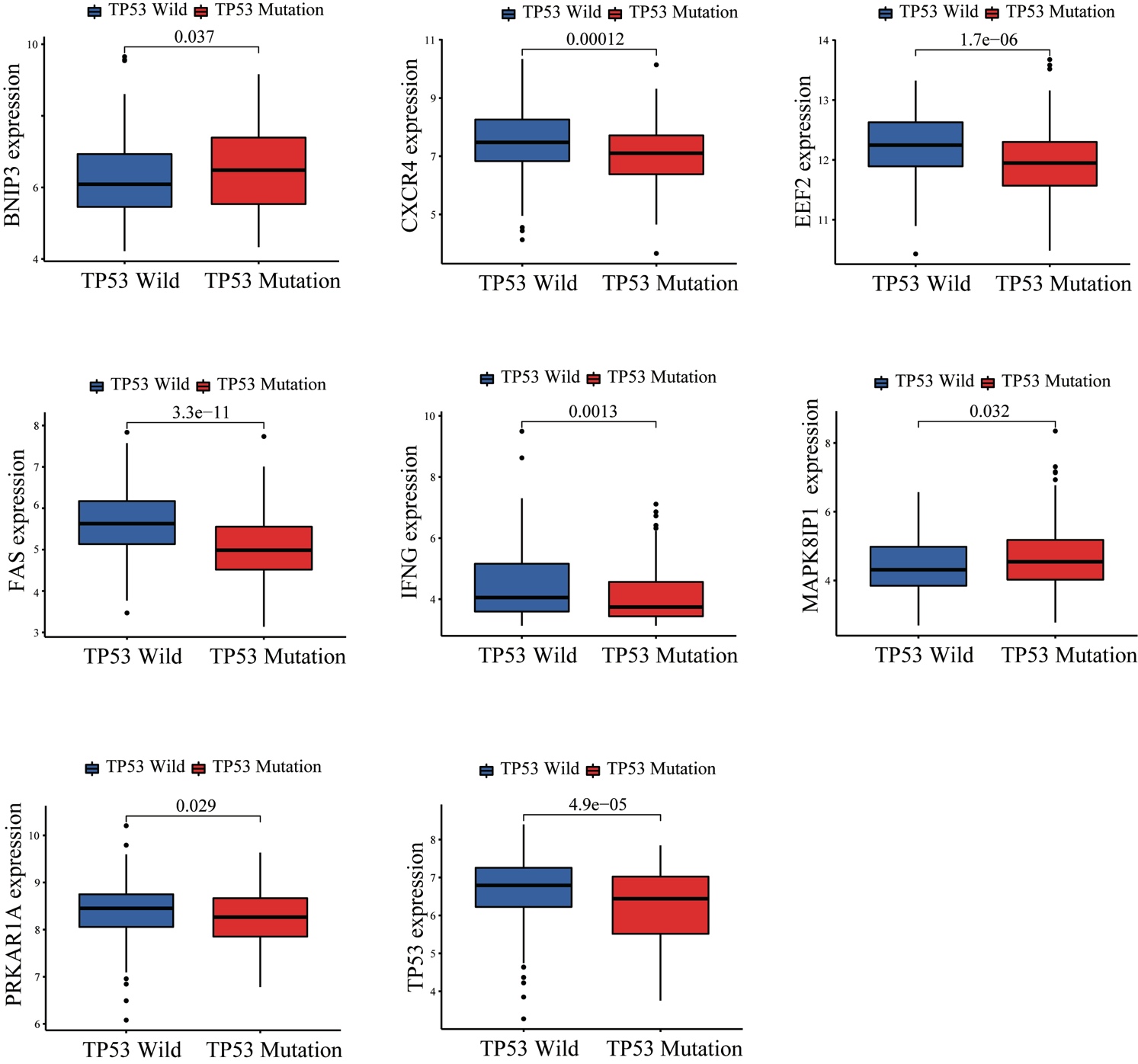


**Figure S1. Analysis of the correlation between ARGs expression level and TP53 mutation status.**


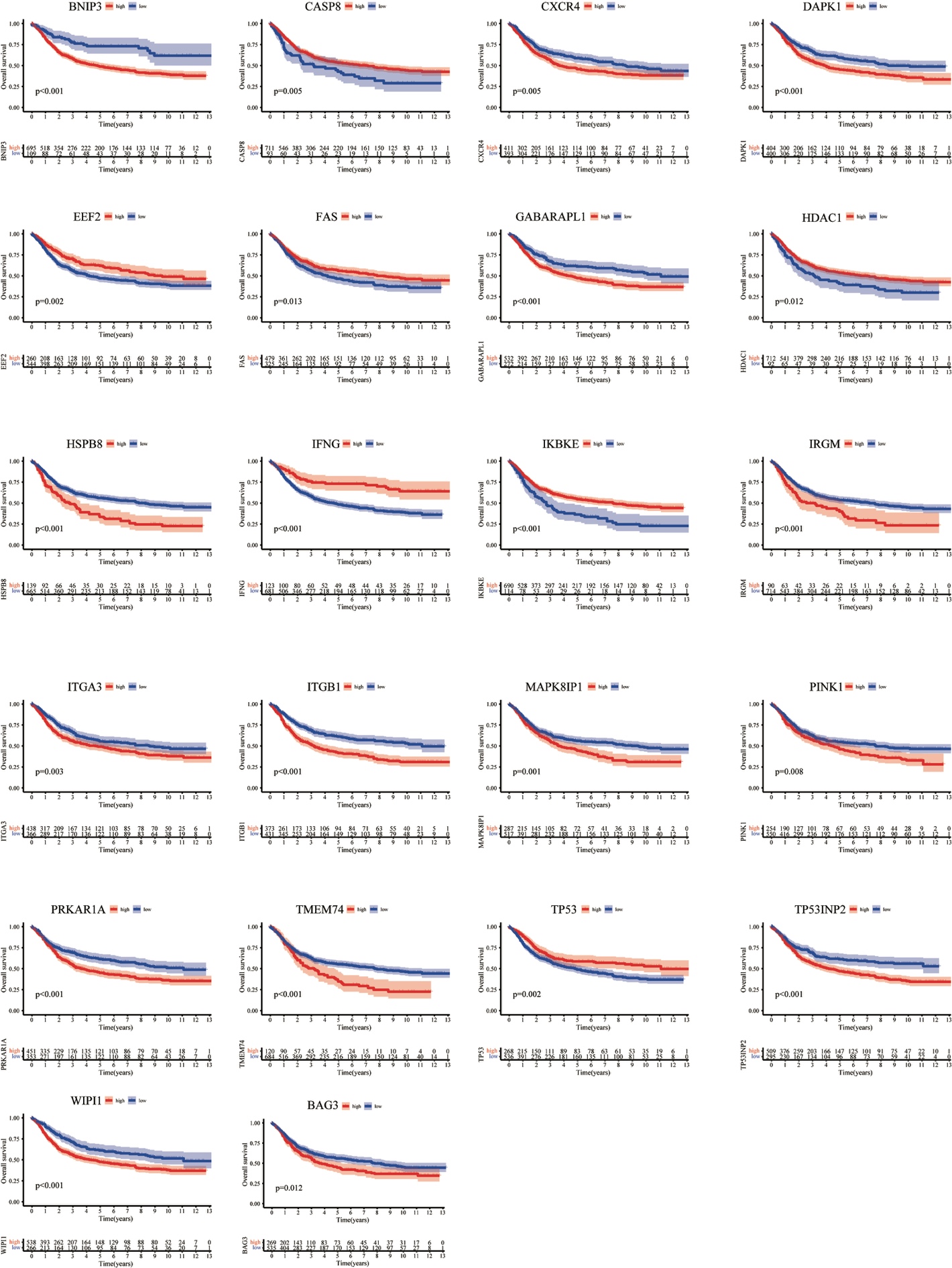


**Figure S2.** **Survival analysis of ARGs in gastric cancer patients.**

**
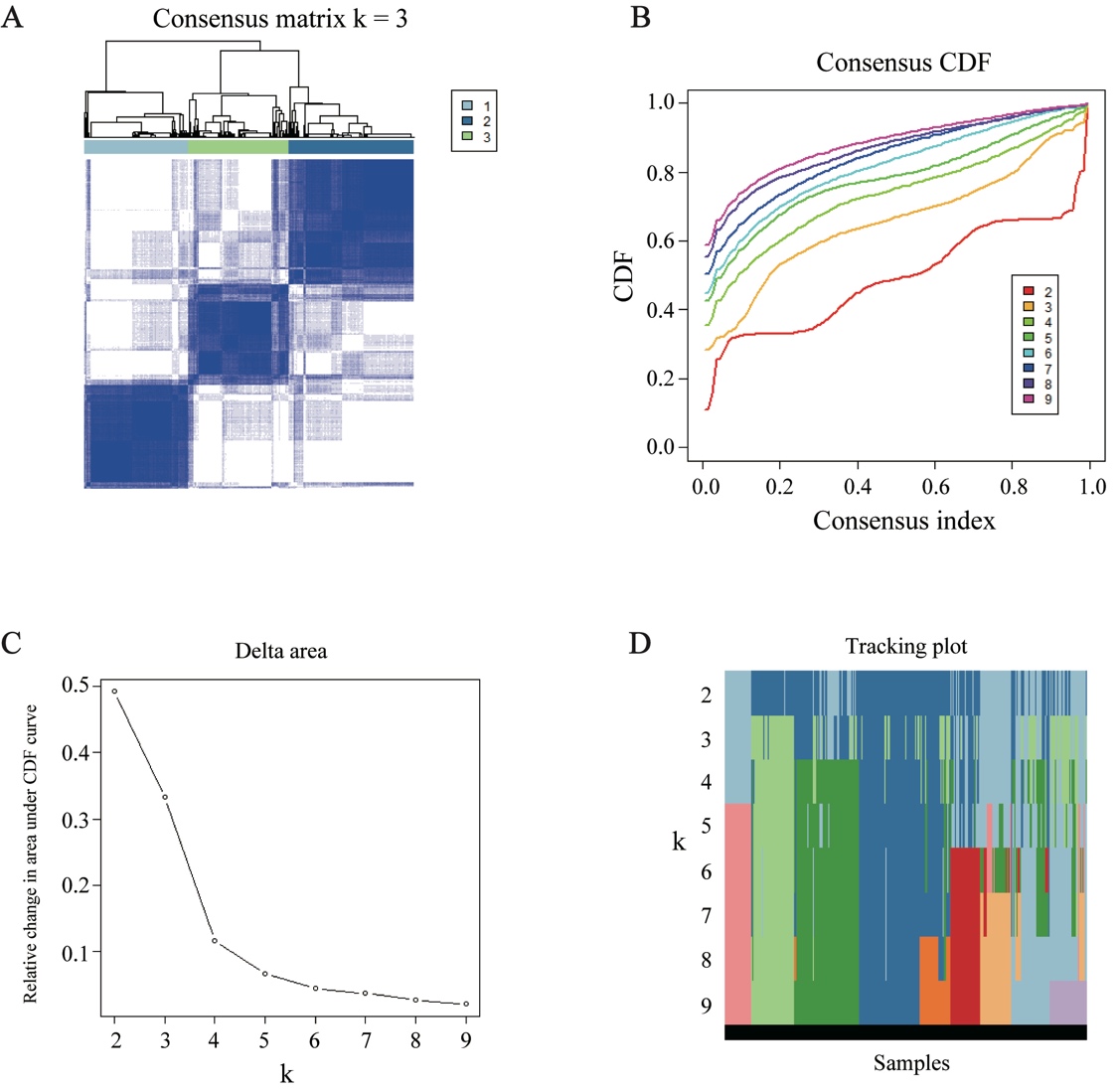
**

**Figure S3.** **Identification of autophagy clusters based on DEGs.** **(A)** Consensus matrix heatmap shows three clusters (k = 3) and their corresponding regions. **(B)** The CDF curves when taking different k values. **(C)** CDF delta area curve of consensus clustering. **(D)** Heatmap of sample clustering under different K values.

**
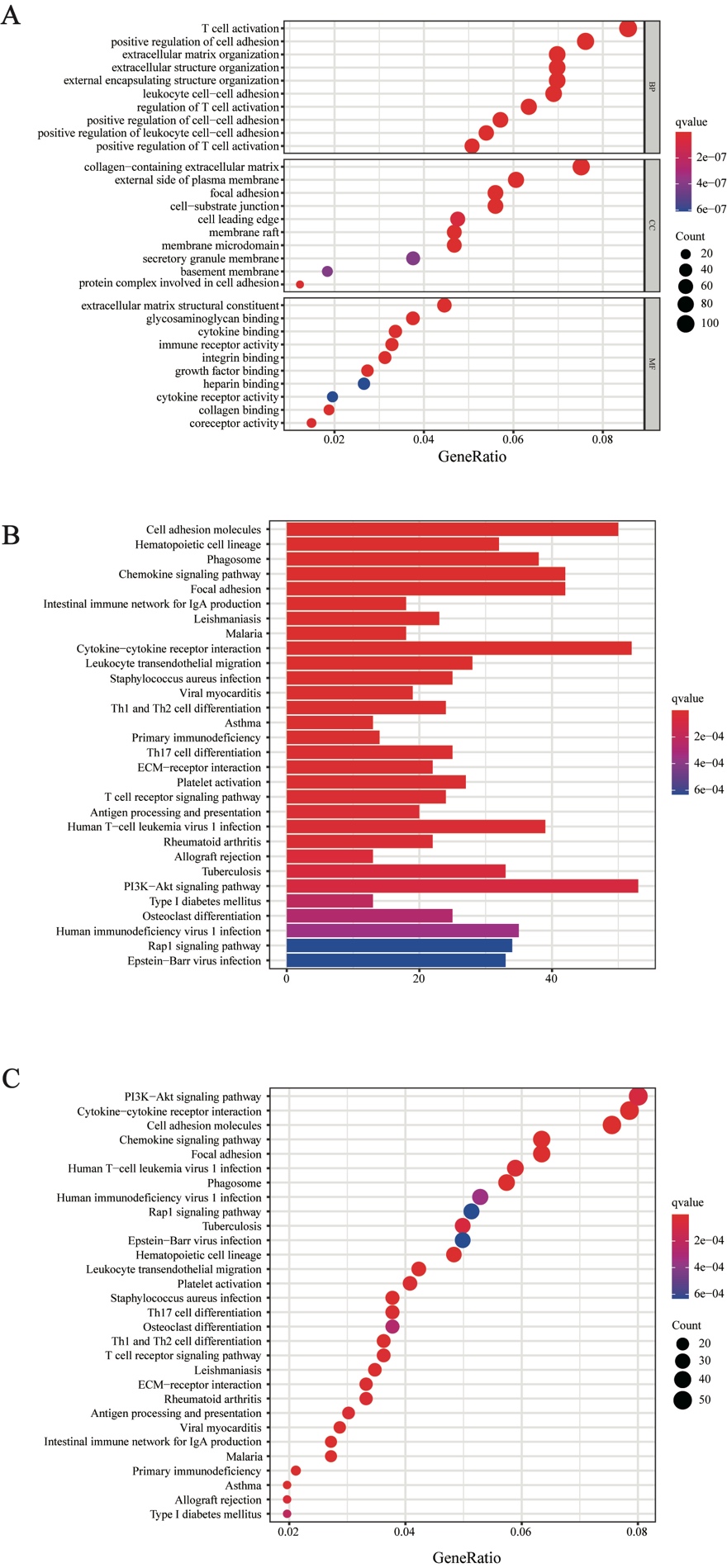
**

**Figure S4. GO enrichment and KEGG enrichment analysis of ARGs.** **(A)** GO enrichment analysis of the overlapping genes. **(B-C)** KEGG enrichment analysis of overlapping genes.


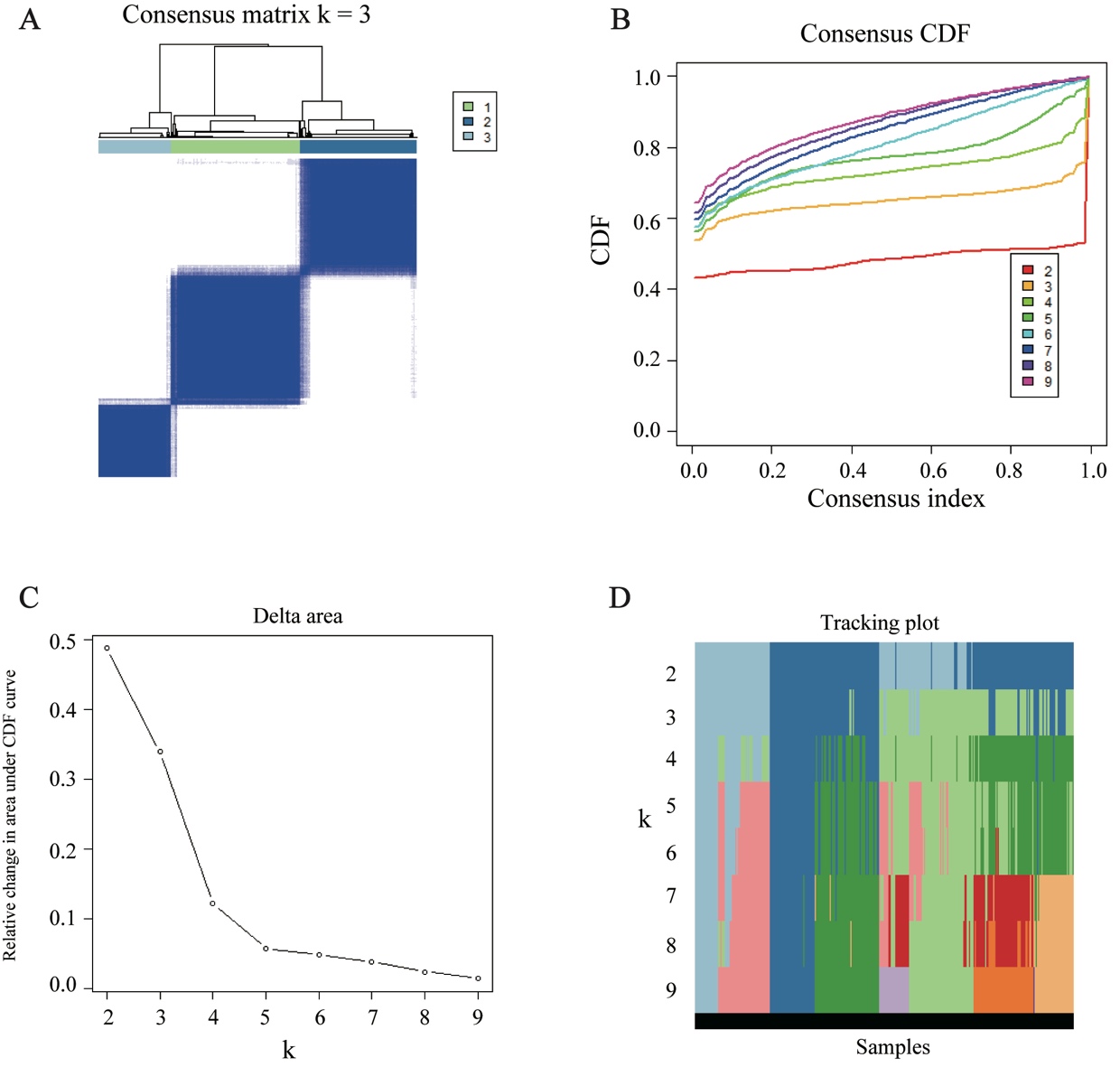


**Figure S5. Identification of gene clusters based on DEGs. (A)** Consensus matrix heatmap shows three clusters (k = 3) and their corresponding regions. **(B)** The CDF curves when taking different k values. **(C)** CDF delta area curve of consensus clustering. **(D)** Heatmap of sample clustering under different K values.
